# The impact of adult attachment on career decision-making in Chinese college students: the chain mediating role of self-differentiation and social support

**DOI:** 10.3389/fpsyg.2025.1531292

**Published:** 2025-04-14

**Authors:** Xiaoqing Wang, Qingli Liu, Biyan Xiao

**Affiliations:** School of Economics and Management, Weifang University of Science and Technology, Weifang, China

**Keywords:** adult attachment, self-differentiation, social support, career decision-making, chain mediating role

## Abstract

**Introduction:**

This study investigates the influence of adult attachment on career decision-making among Chinese university students, focusing on the chain mediation roles of self-differentiation and social support. Grounded in attachment theory and Bowen’s family systems theory, the proposed model “adult attachment → self-differentiation → social support → career decision-making” elucidates how emotional dependency indirectly shapes career choices through psychological maturity and social resource integration, offering novel insights for career counseling.

**Methods:**

Data were collected from 908 students across three universities in Shandong Province using standardized scales: the Experiences in Close Relationships Inventory, Revised Differentiation of Self Inventory, Social Support Rating Scale, and Career Decision Inventory. Structural equation modeling (SEM) tested the hypothesized chain mediation, supplemented by cross-group validation and extreme-group analysis to ensure robustness.

**Results:**

Structural equation modeling revealed that adult attachment indirectly affected career decision-making through self-differentiation and social support (*β* = −0.048, *p* < 0.01), with self-differentiation serving as a partial mediator. Secure attachment enhanced social support acquisition via higher self-differentiation (*β* = 0.368), ultimately improving decision efficacy (*β* = 0.330). A direct path (*β* = −0.059, *p* < 0.05) confirmed attachment’s independent impact. Grade-level differences emerged: mediation effects were pronounced among lower-grade students, while seniors prioritized practical factors.

**Discussion:**

The findings validate an emotion-psychology-resource transformation mechanism, demonstrating how secure attachment optimizes decisions through self-differentiation and social support. However, collectivist cultural contexts attenuated self-differentiation’s standalone effects. The results advocate integrated interventions combining emotional regulation and resource mobilization, proposing stage-specific guidance: psychological empowerment for early undergraduates and practical resource provision for seniors. Limitations include regional sampling bias and scale cultural adaptability; future cross-cultural longitudinal studies are warranted.

## 1 Introduction

Career decision-making represents a complex evaluative process wherein individuals systematically assess available vocational alternatives before committing to an optimal career path (Gati and Saka, 2001). The university period constitutes a pivotal developmental phase for such decisions, with collegiate career choices exerting profound influence on subsequent professional trajectories. This critical life-determining behavior emerges from the dynamic interplay between intrinsic psychological factors and extrinsic social influences. Our investigation specifically examines three fundamental psychosocial determinants: adult attachment patterns, self-differentiation capacity, and social support systems, analyzing their synergistic effects on career decision-making processes.

In interpersonal relationships, adults demonstrate characteristic attachment styles that shape their behavioral tendencies (Bartholomew and Horowitz, 1991). Research identifies three primary attachment orientations: secure, anxious, and avoidant. Those with secure attachments typically demonstrate enhanced utilization of occupational information (Saks and Ashforth, 1997) and greater career decision-making self-efficacy (Hirschi, 2010). Conversely, anxious and avoidant attachment correlates with increased vocational uncertainty and constrained career progression (Leenders et al., 2019).

Self-differentiation, conceptualized as the capacity to preserve autonomous identity within close relationships (Skowron and Friedlander, 1998), represents another crucial psychological determinant. Highly differentiated individuals exhibit superior career clarity, independent evaluation skills, and resilient decision-making when confronting vocational pressures (Middleton, 2017).

The social environment contributes substantially through support networks encompassing emotional sustenance, informational resources, and practical assistance from familial, peer, and professional connections (Cohen and Wills, 1985). Robust support systems facilitate access to critical career resources while buffering decision-related stress, thereby enhancing adaptive capacity during vocational transitions.

While existing studies have established connections between adult attachment and career decision-making, the mediating roles of self-differentiation and social support remain underexplored. The current investigation proposes that adult attachment influences career decisions through a sequential mediation process involving both self-differentiation and social support.

Secure attachment patterns facilitate an optimal equilibrium between interpersonal connectedness and personal autonomy. This balanced dynamic enhances individuals’ capacity to establish appropriate relational boundaries (Shaver and Mikulincer, 2007), thereby strengthening their self-differentiation capabilities. Well-differentiated individuals typically experience greater stability and satisfaction in their close relationships. Furthermore, such high-quality interpersonal connections serve as foundations for developing more comprehensive social support systems (Lakey and Orehek, 2011), which subsequently contribute to more effective career decision-making processes. Strong social support provides emotional comfort, informational guidance, and resource assistance to individuals in career decision-making, and individuals use such support to explore different career options and improve their career decision-making ability (Cohen and Wills, 1985; Di Fabio and Kenny, 2016). Conversely, individuals with anxious and avoidant attachment styles exhibit more anxious and avoidant behaviors due to excessive avoidance or over-dependence on intimate relationships (Beeney et al., 2019); this can make it challenging to find a balance in relationships with self and others (i.e., self-differentiation ability is not sufficiently well-developed). As a consequence, this unbalanced intimacy makes it difficult to obtain help and resources from social relationships through effective communication, which in turn affects career decisions. This study explores this chain mediation process, aiming to provide new theoretical insights into the psychological mechanisms of career decision-making and an empirical basis for career guidance and mental health intervention.

**Figure 1:**
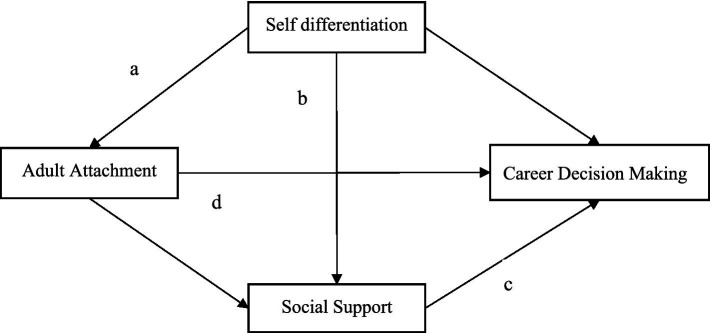
The theoretical framework. Adult attachment improves self-differentiation (Path a), which in turn supports the acquisition of social resources (Path b), leading to enhanced career decision-making capabilities (Path c). Additionally, attachment insecurity directly reduces decision-making effectiveness (Path d).

As shown in Figure 1, the theoretical framework of this study introduces a novel integration of attachment styles, psychological maturity (self-differentiation), and social resources (support networks). By moving beyond traditional single-factor explanations, the framework highlights the interplay between emotional, cognitive, and environmental dimensions, revealing the complex, multi-layered processes that influence career decisions. This model provides a clear intervention strategy for career counseling, centered on emotional regulation, establishing healthy boundaries, and activating support networks.

## 2 Literature review

Existing research has independently explored the roles of adult attachment (Bartholomew and Horowitz, 1991), self-differentiation (Skowron and Friedlander, 1998), and social support (Cohen and Wills, 1985) in career decision-making. However, the interplay among these factors has not been fully investigated. In this study, we integrate attachment theory with Bowen’s family systems theory to develop a chain mediation model, “adult attachment → self-differentiation → social support → career decision-making” (Figure 1). This model emphasizes that adult attachment influences career decisions by fostering emotional independence (self-differentiation) and enhancing the ability to access resources (social support). By offering a comprehensive framework, this study advances our understanding of the psychosocial processes underlying career decision-making and broadens the scope of attachment theory.

### 2.1 The relationship between adult attachment and career decision-making

Attachment theory hypothesizes that high-quality attachment relationships help solve problems related to individual development tasks while providing a stable base for personal development. At present, the relationship between adult attachment and career decision-making has been widely explored, and adult attachment style is thought to affect the behavior patterns of individuals in career choices and job adaptation. Attachment theory has been applied to the study of career decision-making processes, particularly among emerging adults, and studies (e.g., Kvitkovičová et al., 2017) have shown that individuals with secure attachment are more likely to seek support from significant others (e.g., parents, friends, and romantic partners) and thus explore the world around them more confidently. This assertive exploration is based on their mental representations (i.e., their “internal working model”), which suggests that significant others will provide support when needed. Research indicates that securely attached adolescents demonstrate greater propensity for vocational exploration and stronger career commitment. This pattern emerges from the consistent, reliable caregiving experiences that foster well-adjusted self-concepts (Bowlby, 1982). In contrast, those with insecure attachment often restrict their vocational options, constrained by either performance anxiety or relational avoidance (Mikulincer and Shaver, 2007).

Wright (2017) theoretical integration bridges attachment theory with social cognitive career theory (SCCT), creating a more robust model for examining attachment’s role in career development. SCCT’s core components - particularly self-efficacy beliefs, outcome expectations, and goal-setting processes (Lent et al., 1994) - interact with attachment styles to shape career decision-making pathways. Within this synthesized framework, attachment security operates through multiple channels: it enhances perceived self-efficacy regarding career tasks, modifies expectations about potential outcomes, and influences goal selection processes. These mechanisms collectively inform both career counseling interventions and future research directions. While Wright (2017) demonstrated that attachment affects job-seeking behavior via self-efficacy, the study did not examine the contributions of self-differentiation and social support. By contrast, the present model uncovers a more intricate “emotional-to-social” transformation process through chain mediation, offering a more comprehensive framework that overcomes the shortcomings of single-mediator approaches.

### 2.2 The relationship between adult attachment and self-differentiation

Securely attached individuals demonstrate emotional autonomy in close relationships (Mikulincer and Shaver, 2007), which aligns closely with the core feature of self-differentiation—the ability to balance emotion and rationality (Bowen, 1978). Research indicates that individuals with attachment anxiety typically exhibit lower levels of self-differentiation due to excessive emotional dependency (Wang and Fu, 2019). In contrast, those with avoidant attachment display a distinct differentiation pattern: they maintain superficial independence through emotional detachment, yet fall into a state of “pseudo-differentiation,” characterized by overreliance on rational defenses to suppress emotional connections, resulting in rigid interpersonal functioning (Skowron and Schmitt, 2003). Such differentiation deficits may be amplified in collectivist cultures. Cultural contexts significantly shape these psychological dynamics. Within Chinese familial systems, traditional filial piety norms can exacerbate existing attachment patterns - amplifying emotional reliance among anxiously attached individuals while reinforcing behavioral compliance over genuine emotional engagement among avoidant individuals (Li and Kato, 2006).

Emerging evidence reveals a reciprocal relationship between attachment security and self-differentiation. Clinical interventions demonstrate this bidirectional influence: therapeutic approaches that strengthen attachment security (particularly through emotional regulation training) simultaneously promote self-differentiation (Mikulincer and Shaver, 2016). Conversely, as individuals develop greater differentiation capacity, their relational security often improves correspondingly.

Notably, intervention strategies must be tailored to specific attachment styles. Research indicates that anxiously attached individuals benefit most from family systems interventions that restructure dependency patterns, while avoidant individuals show greater improvement through carefully structured group bonding experiences designed to rebuild emotional attunement (Chen et al., 2020). These findings collectively underscore that the attachment-differentiation relationship constitutes a complex, mutually reinforcing system that evolves within particular developmental and sociocultural milieus.

### 2.3 The relationship between self differentiation and career decision-making

Self-differentiation is a core concept in Bowen’s family system theory, which refers to an individual’s ability to remain emotionally independent of the family system. Research by Skowron and Friedlander (1998) shows that in the occupational domain, self-differentiation is related to an individual’s ability to make career decisions. The influence of self-differentiation in career decision-making is mainly reflected in how individuals deal with the emotional and cognitive processes related to career choices. Skowron and Friedlander (1998) also demonstrated a positive correlation between self-differentiation and career decision-making self-efficacy, indicating that better self-differentiation helps to enhance individuals’ self-confidence and efficacy in defining career goals, and individuals with higher levels of self-differentiation show higher autonomy and adaptability in career choice and career switching.

### 2.4 The impact of social support on career decision-making

Social support is considered a critical factor in relieving the stress of career decision-making. Cohen and Wills (1985) propose that it enhances career decision-making capacity by providing emotional comfort, information resources, and practical help. Different types of social support (e.g., emotional, informational, and assessment support) have different effects on career decisions. Cohen and Gati (2021) found that social support has a significant impact on career decision-making difficulties. Social support indirectly influences career decision-making difficulties through enhancing psychological capital and decision-making self-efficacy. The study also demonstrated a positive correlation between social support and career decision-making self-efficacy (overall and its various dimensions; Ng et al., 2010), and subjective support positively predicted the career decision-making self-efficacy of college graduates (Solberg and Ali, 2017).

### 2.5 The mediating role of self-differentiation and social support

Individuals with high levels of self-differentiation are better at establishing healthy interpersonal boundaries (Skowron and Schmitt, 2003), which in turn enables them to more effectively obtain and utilize social support (Lakey and Orehek, 2011). This capability is crucial in career decision-making, as social support can provide informational, emotional, and instrumental resources (Blustein et al., 2008). Another study demonstrates that the facilitative role of self-differentiation in acquiring social support is moderated by cultural specificity. In collectivist cultures, individuals with high self-differentiation excel at employing “boundary negotiation” strategies to obtain support—articulating career needs clearly while preserving familial or group harmony (Wang and Fu, 2019). For instance, Chinese university students proactively engage with family expectations regarding career choices, transforming traditional values into informational resources (e.g., leveraging familial industry experience) rather than adopting unilateral resistance (Li and Kato, 2006). Conversely, those with low self-differentiation, due to emotional fusion or pseudo-independence, often face a support acquisition paradox: over-reliance on single sources (e.g., parental input) or rejection of external assistance, ultimately compromising the quality of support received (Lakey and Orehek, 2011).

### 2.6 The relationship between adult attachment, self-differentiation, social support, and difficulties in career decision-making

Although the independent effects of the above-described variables have been explored, relatively few studies have been conducted on the chain mediating effect of their interactions. Feeney and Collins (2015) propose that intimacy affects the ability to obtain social support and the adaptability of career decisions. Research suggests that secure attachment relationships provide individuals with a “safe base” from which they can more confidently explore the world, including their career choices and career development. This exploration is based on the individual’s mental representations (i.e., “inner working models”) that imply that a significant other person will provide support when needed. In their model, the ability to self-differentiate enables individuals to remain autonomous in relationships while also being able to effectively seek and utilize social support. Improvement in self-differentiation abilities helps individuals better understand their needs and desires when facing career decisions so as to make choices that align better with their interests. In addition, the study also pointed out that the support and care that individuals feel in relationships can indirectly influence the individual’s career decision-making process by promoting self-growth and exploration. This support can come from a partner, family member, friend, or co-worker, who provides the individual with the necessary resources to help them overcome difficulties and seize career opportunities.

Overall, the importance of adult attachment security in career development has been highlighted in prior studies, particularly in career decision-making. The theoretical frameworks on which these studies are based provide valuable insights into how individuals can achieve career advancement through self-differentiation and exploration, supported by intimate relationships. However, empirical research in the field of career development is insufficient. Against this background, this study proposes the hypothesis that adult attachment relationships affect career decision-making not only directly but also indirectly through self-differentiation and social support; it empirically tests this hypothesis.

### 2.7 The theoretical framework of this study

The theoretical framework of this study is presented in Figure 1. Adult attachment not only directly influences career decision-making but also indirectly affects it through the chain path of self-differentiation and social support. Individuals with secure attachment develop effective support networks through high self-differentiation, thereby enhancing decision-making efficacy.

## 3 Hypotheses

Based on the mediation analysis framework developed by Baron and Kenny (1986), both direct and indirect effects can exist simultaneously in a single model. A partial mediation effect is identified when the direct effect decreases but remains significant after accounting for the mediator, whereas full mediation occurs if the direct effect becomes non-significant. In this study, we hypothesize that adult attachment not only directly impacts career decision-making (H1) but also exerts an indirect influence through a chain mediation pathway involving self-differentiation (H5) and social support (H6) (H7). This approach combines attachment theory (Bowlby, 1982) with the social cognitive perspective on career decision-making (Lent et al., 1994), highlighting the multi-dimensional role of emotional traits. The hypotheses are structured around four key aspects: direct effects, single mediation effects, chain mediation effects, and moderating effects.

### 3.1 The hypothesis of direct effect

*H1:* Adult attachment has a direct negative effect on career decision making.

Our theoretical framework posits that adult attachment patterns exert direct effects on collegiate career decision-making processes. This proposition is grounded in observable behavioral patterns: students exhibiting anxious attachment tendencies frequently demonstrate career decision-making difficulties characterized by chronic hesitation and choice paralysis. Similarly, those with avoidant attachment styles often display commitment avoidance behaviors when confronted with career selection tasks. These manifestations stem from fundamental uncertainties regarding vocational pathways and a psychological resistance to making binding career commitments.

### 3.2 The hypothesis of single mediation effect

*H2:* Self-differentiation directly affects career decisions.

Self-differentiation, defined as the capacity to maintain emotional and cognitive autonomy while engaged in interpersonal relationships, plays a pivotal role in vocational decision processes. Drawing upon Bowen’s family systems theory, we propose that university students demonstrating higher self-differentiation capacities exhibit three distinct advantages in career decision-making: (1) greater independence in vocational choice determination, (2) enhanced emotional regulation when confronting career-related stressors, and (3) improved adaptive functioning when navigating the uncertainties inherent in career transitions

*H3:* Social support directly influences career decisions.

The career decision-making process among university students is significantly influenced by multidimensional social support systems, encompassing emotional sustenance, informational guidance, and tangible resources. Empirical evidence suggests these support networks serve three critical functions in vocational development: (1) facilitating access to relevant labor market information and professional networks, (2) enhancing the quality of career-related decision-making through expert guidance, and (3) mitigating psychological distress associated with career uncertainty. We postulate that robust social support structures enable students to navigate career transitions more effectively by providing both practical resources and psychological buffers during this developmental milestone.

*H4:* There is a correlation between adult attachment, self-differentiation, social support, and career decision-making.

Our theoretical framework posits dynamic interrelationships among three key psychological constructs: adult attachment styles, self-differentiation capacity, and social support systems. These factors operate synergistically to shape collegiate career decision-making processes. Specifically, students exhibiting secure attachment patterns demonstrate two distinct advantages: (1) greater propensity for developing mature self-differentiation skills, and (2) enhanced ability to cultivate and utilize social support networks. This psychological configuration creates an optimal foundation for making informed, adaptive career choices during the critical transition from academia to professional life.

*H5:* Self-differentiation partially mediates the effect of adult attachment on career decision-making.

Our theoretical model proposes self-differentiation as a critical psychological mechanism through which adult attachment patterns influence career decision-making competencies. Specifically, we posit that an individual’s characteristic attachment style shapes their capacity for self-differentiation, which in turn affects their vocational decision-making processes. This mediating pathway suggests that the effects of early relational experiences on career choices operate, at least partially, through the development of emotional and cognitive autonomy in interpersonal contexts.

*H6:* Social support partially mediates the effect of adult attachment on career decision-making.

Building upon established theoretical frameworks, we propose that social support serves as a crucial intermediary in the association between adult attachment patterns and career decision-making outcomes. The model suggests that individuals’ characteristic attachment orientations systematically influence both the availability and effective utilization of social support resources. These variations in social support accessibility and engagement subsequently contribute to differences in career decision-making processes and outcomes.

### 3.3 The chain mediation hypothesis

*H7:* Self-differentiation and social support mediate in a chain between adult attachment and career decision-making.

We hypothesize that self-differentiation and social support play a chain mediating role between adult attachment and career decision-making. Attachment style may first affect self-differentiation, which then affects the way individuals obtain social support, and ultimately, these factors together influence career decisions.

### 3.4 The moderation effect hypothesis

*H8:* The structural relationships between variables vary by gender, grade, subject, and educational level.

In the context of adult attachment, gender may influence an individual’s behavior patterns and coping strategies in intimate relationships, which in turn can influence career decisions. College students may face different career development tasks and challenges at different grade levels, which may affect their career decision-making process. Students from different academic backgrounds may have different career interests and paths. Disciplines may influence students’ reception and processing of career information, as well as their expectations and goals for future careers. The level of education may influence an individual’s career aspirations and choices. For example, undergraduates may have higher career expectations than junior college students or have more clear research directions and career goals.

## 4 Methods

### 4.1 Participants

In order to control for the impact of sampling universities at different levels, we selected three comprehensive universities in Shandong Province (namely W University, Q University, and J University), which are roughly equivalent in terms of geographical location, education level, and faculty quality. These universities offer a diverse range of professional education and rich campus cultural activities, providing students with a suitable environment for learning and growth. All three universities are located in second-tier cities, and their employment rates have remained above 85%. Data collection was conducted from 10 to 22 March 2024. A total of 1,000 people were surveyed with the assistance of teachers and students from the surveyed institutions; however, due to 92 invalid questionnaires, data from 908 participants were analyzed.

Table 1 shows the background characteristics of the participants. These variables can provide richer background information for the research, helping us better understand how adult attachment, self-differentiation, and social support affect the career decisions of Chinese college students. By analyzing these variables, we can explore the differences between different groups in the career decision-making process, as well as the underlying reasons behind these differences.

**Table 1:** The basic characteristics of the study participants.

Variable	Value	*N*	%
Gender	Male	334	36.8
	Female	574	63.2
Grade	Freshman	326	35.9
	Sophomore	359	39.5
	Junior	161	17.7
	Senior	62	6.8
Subject area	University of Liberal Arts	506	55.7
	University of Science	305	33.6
	Medical University	97	10.6
Educational level	Undergraduate students	521	57.4
	College students	278	30.6
	Adult education students	109	12.0
Total		908	100

### 4.2 Research tools

#### 4.2.1 Adult attachment

We used the Experiences in Close Relationships Inventory Intimate Relationship Experiences Scale, jointly developed by Fraley and Shaver (2000). The scale contains a total of 36 items, which are composed of two sub-factors: attachment avoidance and attachment anxiety. Each sub-factor consists of 18 items. Avoidant attachment is described as “the tendency to distrust the other person and maintain an emotional distance from the other person,” while attachment anxiety is described as “a lack of information about the emotional utility of the intimate other, a strong desire to be close to the intimate other, and a feeling of uneasy emotions.” The higher the score on the questionnaire, the higher the level of unstable attachment. A 7-point Likert scale was used (1 = Strongly Disagree, 7 = Completely Agree). The adult Cronbach’s alpha coefficient for this study was 0.822, 0.808 for attachment avoidance, and 0.747 for attachment anxiety.

#### 4.2.2 Self-differentiation

The Differentiation of Self Inventory, developed by Skowron and Schmitt (2003), was used. The scale consists of 21 items in four dimensions: qualitative response (ER), emotional isolation (EC), and integration with others (FO). ER reflects an individual’s sensitivity to stimuli in the environment and emotional stability, while EC reflects an individual’s aversion or fear of intimate experiences and the emotional tendency to defend against others during interactions with others. FO is the degree of an individual’s emotional will and integration with others. The higher the scores, the higher the level of self-differentiation. Items were answered on a 6-point Likert scale (1 = not at all compliant, 6 = completely compliant). In this study, the Cronbach’s alpha coefficient of the scale was 0.836 (subscales: ER = 0.728, EC = 0.645, and FO = 0.780).The Emotional Cutoff (EC) subscale of the self-differentiation scale yielded a Cronbach’s *α* of 0.645, which is slightly lower than the conventional benchmark of 0.70 (Kline, 2015). Several factors may explain this outcome. First, cultural context plays a role: the EC subscale assesses avoidance tendencies in close relationships, but Chinese cultural norms prioritize emotional restraint (Li and Kato, 2006), potentially leading to similar responses and reduced item discrimination. Second, the limited number of items in the EC subscale (only five) increases susceptibility to random error (Tavakol and Dennick, 2011). Third, the sample’s homogeneity—focusing exclusively on college students with relatively uniform emotional expression patterns—may have further constrained variability. Despite the lower reliability of the EC subscale, the overall self-differentiation scale met acceptable reliability standards (α > 0.70). To enhance reliability, future research could consider expanding the number of items or refining the wording to better align with cultural contexts.

#### 4.2.3 Social support

The Multidimensional Scale of Perceived Social Support (MSPSS) developed by Zimet et al. (1988) was used. The 12-item scale consists of three subscales: family support, friend support, and significant other support. Each with 4 items. Each item is rated using a 7-point scale ranging from 1 (strongly disagree) to 7 (strongly agree). The total score is obtained by summing the scores of all the items, a higher score means a higher level of social support. The Cronbach’s alpha coefficient in this study was 0.719.

#### 4.2.4 Career decisions

The Career Decision Difficulties Questionnaire, developed by Gati et al. (1996) was used. The 16-item scale includes four subscales, including career information exploration, career self-exploration, career planning exploration, and career goal confidence. Career information exploration is the individual’s active acquisition of information through multiple channels. Career self-exploration is the individual’s initiative to understand himself through multiple channels, while career planning exploration is the individual’s active pursuit of information about career decision-making and planning, and career goal determination is the embodiment of the individual’s confidence in the career development goal. The higher the score obtained, the more proactive and confident one is in career decisions. A 5-point Likert level scale (1 = not at all, 5 = completely compliant) was used. In this study, the Cronbach coefficient of the scale was 0.858, while it was 0.822 for career information exploration, 0.725 for career self-exploration, 0.747 for career plan exploration, and 0.811 for career goal confidence.

### 4.3 Data analysis

In order to understand the characteristics of adult attachment, self-differentiation, social support, and career decision-making of Chinese college students, the descriptive statistics of each variable were calculated. In order to investigate the relationship between adult attachment, self-differentiation, social support, and career decision-making, Pearson correlation analysis was conducted. In order to investigate the structural relationship of the variables, we used a maximum likelihood (ML) structural equation model (SEM). In this study, the kernel function method was used to convert discrete variables into continuous variables before using the SEM. To validate the statistical robustness of the model and to reveal its theoretical applicability and practical significance across different groups, this study conducted an extreme-group analysis. Based on the total scores of the adult attachment variable, the sample was divided into a high attachment insecurity group (the top 20% of students, totaling 182 individuals), a low attachment insecurity group (the bottom 30% of students, totaling 272 individuals), and a moderate group (the remaining 50% of students, totaling 454 individuals). The model was fitted separately for the high and low scoring groups to verify whether the core pathways remained stable among the extreme groups in terms of attachment levels.

## 5 Results

### 5.1 Common method bias analysis

The Harman one-factor method was used to test for common method bias. The results show that a total of 23 factors had eigenroot values greater than 1, and the first factor explained 11.23% of the total variation (below the cut-off value of 40%), so the effect of common method bias was negligible.

### 5.2 Descriptive statistics

The means (M) and standard deviations (SD) for each variable and its sub-dimensions are presented in Table 2. The average score for adult attachment among Chinese university students was 3.584, with an SD of 0.664. The mean score for self-differentiation was 3.434, with an SD of 0.614. For social support, the average score was 2.78, with an SD of 0.46. The mean score for career decision-making was 3.342, with an SD of 0.63.

**Table 2:** Descriptive statistics for variables and subdimensions.

Variable	*M* (SD)	Subdimensions	*M* (SD)
Adult attachment	3.584 (0.664)	Anxiety	3.488 (0.737)
		Avoidance	3.680 (0.795)
Self-differentiation	3.343 (0.614)	Emotional reactivity (ER)	3.204 (0.891)
		Emotional cutoff (EC)	3.823 (0.885)
		Fusion with others (FO)	3.310 (0.850)
Social support	2.780 (0.460)	Family support	3.090 (0.500)
		Friend support	2.670 (0.740)
		Significant other support	2.560 (0.560)
Career decision-making	3.342 (0.636)	Career information exploration	3.426 (0.711)
		Career self-exploration	3.435 (0.720)
		Career planning exploration	3.123 (0.864)
		Career goal certainty	3.309 (0.759)

### 5.3 Correlation analysis

The results of the correlation analysis among the variables are shown in Table 3. Adult attachment is negatively correlated with career decision-making (*r* = −0.262**), self-differentiation (*r* = −0.481**), and social support (*r* = −0.252**). Social support is positively correlated with career decision-making (*r* = 0.241**) and self-differentiation (*r* = 0.24**).

**Table 3:** Correlation coefficients between variables.

Variable	Adult Attachment	Attachment Avoidance	Attachment Anxiety	Self-Differentiation	Emotional Reactivity	Emotional Cutoff	Fusion with Others	Social Support	Family Support	Friend Support	Significant Others	Career Decision-Making	Information Exploration	Self-Exploration	Planning Exploration	Goal Exploration
1	1															
2	0.636**	1														
3	0.843	0.104**	1													
4	−0.481**	−0.275**	−0.535**	1												
5	−0.273**	−0.025	−0.376**	0.775*	1											
6	−0.454	−0.270**	−0.391**	0.701**	0.478**	1										
7	−0.463	−0.053	−0.547**	0.895**	0.573**	0.541**	1									
8	−0.252**	−0.221**	−0.166**	0.237**	0.136**	0.278**	0.155**	1								
9	−0.224**	−0.191**	−0.152**	0.222**	0.138**	0.225**	0.139**	0.863**	1							
10	−0.206**	−0.150	−0.158**	0.165**	0.108**	0.182**	0.029**	0.710**	0.381**	1						
11	−0.111**	−0.147	−0.038	0.119**	0.030	0.222**	0.063	0.626**	0.335**	0.275**	1					
12	−0.262**	−0.048	0.020	0.131**	−0.022	0.036	0.017	0.241**	0.267**	0.069**	0.163**	1				
13	−0.114	−0.057	−0.046	0.156**	0.040	0.075*	0.077*	0.212**	0.249**	0.055	0.125**	0.881**	1			
14	0.242*	−0.091**	0.072*	−0.046	−0.096**	0.024	−0.104	0.199**	0.225**	0.034	0.156**	0.820**	0.662**	1		
15	0.036	0.021	0.033	0.118**	−0.007	0.006	0.034	0.160**	0.163**	0.060	0.118**	0.792**	0.560**	0.515**	1	
16	−0.037	−0.024	0.024	0.222*	−0.022	0.006	0.035	0.233**	0.251**	0.084*	0.151***	0.868**	0.676**	0.585**	0.659**	1

注:*p < 0.05, **p < 0.01, ***p < 0.001. 表中, 1 = Adult Attachment; 2 = Attachment Avoidance; 3 = Attachment Anxiety; 4 = Self-Differentiation; 5 = Emotional Reactivity; 6 = Emotional Cutoff; 7 = Fusion with Others; 8 = Social Support; 9 = Family Support; 10 = Friend Support; 11 = Significant Others; 12 = Career Decision-Making; 13 = Information Exploration; 14 = Self-Exploration; 15 = Planning Exploration; 16 = Goal Exploration.

### 5.4 Hypothesis testing based on SEM: fit analysis of structural relationships

**Figure 2:**
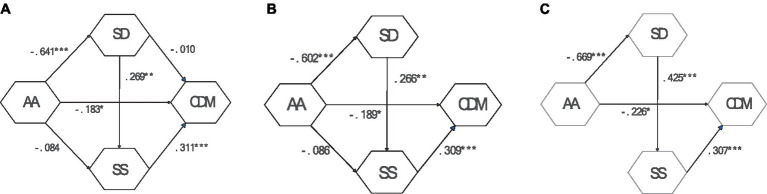
Research model and revised models **(A)** the research model, **(B)** the revised model 1, and **(C)** the revised model 2. *p < 0.05, **p < 0.01, ***p < 0.001. AA: Adult attachment; SD: Self-differentiation; CDM: Career decision-making; SS: Social support.

To better validate the structural relationships among adult attachment, self-differentiation, social support, and career decision-making difficulties, this study designed a research model. The research model (Model A) demonstrated good fit indices:χ^2^ = 205.235, df = 48, *p* < 0.001, ECVI = 0.319, TLI = 0.907, CFI = 0.932, RMSEA = 0.060 (see Table 4). However, in the research model, the direct path from self-differentiation to career decision-making was not significant. Therefore, the first revision was made by removing this path, resulting in Model B. Additionally, the direct path from adult attachment to social support was also not significant, leading to a second revision, which produced Model C. Figure 2 illustrates the specific pathways of the three models. Both the initial research model (Model A) and the revised models (Model B and Model C) exhibited good fit indices (Table 4). Since Model A and the revised models (Model B and Model C) are nested, a difference test was conducted to compare their fit indices. The results showed significant differences between Model A and the revised models, with Model C demonstrating the most ideal fit:χ^2^ = 211.955, df = 50, *p* < 0.001, TLI = 0.911, CFI = 0.936, RMSEA =0.057 (Table 4). Consequently, Model C was selected as the final model, representing a chain mediation model: “Adult Attachment → Self-Differentiation → Social Support → Career Decision-Making.”

**Table 4:** Comparison of the fit of the study model with the revised model.

Model	*χ^2^*	*df*	⊿*χ*^2^(*df*)	ECVI	TLI	CFI	RMSEA
a. Research model	205.24***	48		0.319	0.907	0.932	0.060
b. Revised model 1	206.41***	49	1.18(1)	0.318	0.908	0.932	0.060
c. Revised model 2	211.96***	50	6.72(2)	0.316	0.911	0.936	0.057

***p < 0.001.

### 5.5 Cross-validation test results

To further evaluate the model’s predictive power and ensure its robustness, a cross-validation test was conducted. A randomly selected sample of 423 participants was used for structural model fit analysis, yielding good fit indices: χ^2^ = 106.679, df = 50, p < 0.001, TLI = 0.932, CFI = 0.948, RMSEA = 0.050. These results indicate that the model maintains good fit and stability.

### 5.6 The extreme-group analysis

The extreme-group analysis results showed that the model fit was good for the high attachment group (χ^2^/df = 2.18, RMSEA = 0.053, CFI = 0.928, TLI = 0.910), while the model fit for the low attachment group was even better (χ^2^/df = 1.89, RMSEA = 0.045, CFI = 0.951, TLI = 0.938). These results indicate that the chain mediation model demonstrates stability across different attachment level groups, supporting the generalizability of the study’s conclusions.

### 5.7 Sensitivity analysis: impact of removing the EC subscale on model results

Additionally, to examine the potential impact of the low reliability of the EC subscale of self-differentiation on the results, this study removed the EC dimension and recalculated the self-differentiation score (retaining only the ER and FO dimensions), followed by repeating the SEM analysis. The results indicated that the model fit remained good (χ^2^/df = 2.14, RMSEA = 0.051, CFI = 0.932, TLI = 0.915).

### 5.8 Analysis of mediation effects

To test the partial mediation hypothesis, the Bootstrap method (with 1,000 resamples) was employed to examine both direct and indirect effects. If the total effect (direct + indirect) is significant and the direct path remains significant after including the mediating variables, it supports partial mediation. Conversely, if the direct path becomes non-significant, it supports full mediation (Preacher and Hayes, 2008).the results are shown in Table 5. The direct effect was shown on the path from adult attachment to self-differentiation (−0.398, *p* < 0.01). There were both indirect effects (−0.048, *p* < 0.01) and direct effects (−0.059, *p* < 0.05) on the path from adult attachment to career decision-making. There was a direct effect on the pathway from self-differentiation to social support (0.368, *p* < 0.01) and from social support to career decision-making (0.330, *p* < 0.01). Finally, in the final model, the bicontinuum relationship between self-differentiation and social support was analyzed for the path from adult attachment to future decision-making; the results showed that in the path from adult attachment → self-differentiation → social support → career decision-making, the lower and upper limits of bootstrapping did not contain zero, and the bicontinuous mediating effect (−0.048, *p* < 0.01) was significant. Specifically, the direct effect of adult attachment on career decision-making was significant, while the chain mediation effect through self-differentiation and social support was also significant, supporting the coexistence of H1 and H7. This result aligns with the partial mediation model, indicating that attachment insecurity not only directly influences career decision-making but also exerts an indirect effect through psychological and social mechanisms. Thus, several hypotheses (H1, H3, H5, H6, and H7) were supported, while H2 was rejected.

**Table 5:** Mediation effect analysis of self-differentiation and social support.

Path	Indirect effects	Direct effect	Total effect
AA → SD		−0.398**	−0.398**
AA → SD → SS → CDM	−0.048**		−0.048**
AA → CDM	−0.048**	−0.059*	−0.010*
AA → SD → SS	−0.146**		−0.146**
SD → SS		0.368**	0.368**
SS → CDM		0.330**	0.330**

***p < 0.001, **p < 0.01, *p < 0.05. AA, Adult attachment; SD, Self-differentiation; CDM, Career decision-making; SS, Social support.

### 5.9 Comparison of differences in structural relationships in demographic contexts

In order to observe whether the structural relationships between variables vary according to the demographic characteristics of the participating college students (e.g., gender, grade, subject type, educational level), a multi-group analysis was carried out using SEM. The results showed that there were no differences in the structural relationship between the variables of different genders, educational levels, and subject categories, but there were significant differences in the structural relationships between the variables of different grades. In the pathway from adult attachment → self-differentiation → social support → career decision-making, the lower and upper limits of bootstrapping in the Freshman, Sophomore, and Junior groups did not include zero (Freshman, −0.028, *p* < 0.05; Sophomore, −0.059, *p* < 0.001; Junior, 0.170, *p* < 0.01), but there was no significant indirect effect in the Senior group. Regarding H8, the hypothesis of differences in structural relationships in the dimension of grade level was accepted, and the hypothesis of differences in the dimensions of gender, educational level, and subject area was rejected.

## 6 Discussion

### 6.1 H7 supported: Self-differentiation and social support mediate in a chain between adult attachment and career decision-making

This study validates the explanatory power of the chain mediation model “Adult Attachment → Self-Differentiation → Social Support → Career Decision-Making.” Individuals with secure attachment develop effective support networks through high self-differentiation, thereby enhancing their decision-making capabilities. This finding aligns with Bowen’s (1978) research, which posits that psychological maturity is the core hub for resource transformation, while also extending the application of attachment theory to the career domain. Specifically, emotional security not only promotes relational health but also facilitates career development through the chain of differentiation and support.

Compared to traditional social cognitive career theory (Social Cognitive Career Theory, SCCT), which emphasizes the direct influence of self-efficacy, outcome expectations, and goal setting on career decision-making (Lent et al., 2000), this study integrates emotional foundations into the core of the decision-making mechanism. The proposed three-stage mechanism—"emotional foundation, psychological transformation, and resource integration”—provides a new paradigm for understanding the long-term effects of attachment.

In the emotional foundation stage, secure attachment provides individuals with emotional stability (Bowlby, 1982), reducing anxiety during career decision-making. In the psychological transformation stage, self-differentiation balances emotions and rationality (Bowen, 1978), enhancing the ability to identify and utilize resources. In the resource integration stage, social support offers decision-making information and emotional encouragement (Cohen and Wills, 1985), addressing the issue of insufficient decision-making resources. This chain mediation effect reveals a complex “psychological-social” transformation process, where self-differentiation requires social support to achieve resource conversion.

The collectivist cultural context in China appears to amplify the significance of this mediated pathway, where social support networks play a more prominent role than purely autonomous decision-making processes. This phenomenon reflects a relational paradigm in career development, wherein individuals strategically mobilize decision-making resources through social channels rather than relying solely on independent judgment. Notably, those with well-developed self-differentiation capabilities demonstrate superior ability to critically evaluate and effectively leverage authoritative support systems, resulting in more optimal career decision outcomes. These observations align with previous findings from cultural studies of career development in China (Wang and Fu, 2019).

Robust methodological approaches, including cross-validation procedures and extreme-group comparisons, substantiate the model’s predictive validity and theoretical coherence. The results demonstrate consistent applicability across diverse attachment profiles, with insecure attachment consistently emerging as a negative predictor of career decision-making competence regardless of subgroup characteristics (Wang and Fu, 2019). This pattern suggests that the interplay between attachment security, self-differentiation, and social support represents a fundamental psychological mechanism underlying career decision processes in the Chinese cultural context.

### 6.2 H1 supported: Adult attachment has a direct negative effect on career decision making

Bowlby’s (1982) attachment theory provides a foundational framework for understanding how adult attachment directly shapes career decision-making processes. The current findings extend this theoretical perspective by demonstrating that insecure attachment patterns impair vocational choices through dual mechanisms: immediate affective interference with rational decision-making and more enduring cognitive distortions about career possibilities. This direct influence model contrasts with Blustein et al.’s (2008) mediation model, a discrepancy that may reflect developmental specificities–university students’ career decisions appear particularly susceptible to emotional regulation challenges during this transitional life stage.

The empirical evidence reveals distinct decision-making patterns across attachment styles. Individuals with anxious attachment tend to exhibit excessive reassurance-seeking behaviors in career choices, making their vocational decisions contingent upon external validation (Mikulincer and Shaver, 2007). This dependence frequently manifests as decision paralysis when confronting occupational uncertainty. Conversely, those with avoidant attachment typically withdraw from career support systems (Berger and McAdams, 2011), often neglecting valuable occupational resources due to discomfort with help-seeking behaviors, which subsequently creates information deficits that compromise decision quality.

In contrast, secure attachment serves as a protective factor that fosters adaptive career efficacy (Bandura, 1997). Securely attached individuals demonstrate three key advantages in career decision-making: they approach vocational exploration with greater confidence, exhibit higher tolerance for career-related ambiguity, and maintain an optimal balance between autonomous judgment and appropriate support-seeking. These findings collectively underscore how attachment security fundamentally scaffolds career decision-making competence through integrated emotional and cognitive channels.

The present research contributes to attachment theory by elucidating the complex interplay between emotional regulation and vocational decision processes during emerging adulthood. The tripartite model developed through this investigation provides a nuanced understanding of how different attachment orientations shape distinct career decision-making patterns, with particular relevance for university students navigating the transition from academic to professional life.

A second influence is information processing and problem-solving. Individuals with insecure attachments may employ more negative strategies (e.g., avoidance or procrastination) when faced with career decisions, while individuals with secure attachments may be more inclined to actively seek solutions (Hirschi, 2010).

### 6.3 H2 not supported: Self-differentiation has no direct effect on career decision-making

The direct effect of self-differentiation on career decision-making was not significant. This suggests that self-differentiation influences the decision-making process only through the mediation of social support, rather than exerting an independent effect. This finding challenges Bowen’s (1978) theory of self-differentiation, which emphasizes the direct impact of psychological independence on decision-making. The discrepancy may stem from cultural factors as a key moderating variable. The sample in this study was drawn from China, where decision-making is heavily influenced by familial, communal, and collectivist values, requiring individuals to rely on external support networks for validation (Li and Kato, 2006). Thus, self-differentiation must operate through social support to affect decision-making.

### 6.4 H3 supported: Social support directly influences career decisions

Social support directly and positively influences career decision-making. This result aligns with Cohen and Wills’ (1985) proposition that social support networks directly enhance decision-making capacity and confidence through emotional support, informational resources, and instrumental assistance. It is also consistent with findings by Wang and Fu (2019). Social support not only indirectly facilitates decision-making by alleviating anxiety but also demonstrates a direct effect, indicating that social support networks themselves serve as critical resources for career decisions. For instance, informational support (e.g., direct guidance from career mentors) can reduce time spent on information searches, while emotional support (e.g., encouragement from family and friends) bolsters decision-making courage. Such immediate resource inputs operate independently of self-differentiation, highlighting the independent value of social support (Lent et al., 2000).

Although social support mediates the chain pathway “self-differentiation → social support → career decision-making,” its direct effect on career decision-making remains significant. This suggests that even when individuals lack sufficient self-differentiation, external social support can still improve decision-making quality. This finding expands traditional mediation models by emphasizing that, in collectivist cultures, external support may partially compensate for deficits in individual psychological resources.

### 6.5 H4 supported: There is a correlation between adult attachment, self-differentiation, social support, and career decision-making

Significant correlations were found among the four variables: adult attachment, self-differentiation, social support, and career decision-making. The negative correlation between adult attachment and career decision-making indicates that higher levels of attachment anxiety and insecurity are associated with greater obstacles in career decision-making, consistent with Bowlby’s (1982) attachment theory. The positive correlation between self-differentiation and career decision-making suggests that higher self-differentiation is linked to stronger decision-making abilities. However, the weak correlation between these variables may stem from China’s collectivist cultural context, where individuals prioritize familial or societal expectations over independent cognition during decision-making (Li and Kato, 2006). The positive correlation between social support and career decision-making highlights the compensatory role of social resources, with greater support reducing decision-making barriers. This aligns with Lent et al., (1994) social cognitive theory, emphasizing the independent contribution of external social resources to individual decisions.

The negative correlations between adult attachment and self-differentiation, as well as adult attachment and social support, reveal that higher attachment anxiety and avoidance correspond to lower self-differentiation and diminished access to relational resources. This dual limitation on psychological and social resources validates Bowen’s (1978) findings that individuals with high attachment anxiety or avoidance struggle to develop emotional independence. It also supports Lakey and Orehek’s (2011) conclusion that such individuals face challenges in acquiring external social support. The positive correlation between self-differentiation and social support confirms that higher self-differentiation facilitates access to social resources, reinforcing the core assumption of Bowen’s family systems theory: psychological maturity forms the foundation for healthy relationships (Skowron and Schmitt, 2003).

### 6.6 H5 supported: Self-differentiation partially mediates the effect of adult attachment on career decision-making

Self-differentiation partially mediates the relationship between adult attachment and career decision-making. Attachment anxiety and avoidance weaken self-differentiation, thereby impairing career decision-making. The current findings reveal important nuances in how attachment characteristics influence career decision-making processes. The lack of a direct effect of self-differentiation suggests that attachment traits likely operate through multiple pathways, some of which remain to be fully explored. This partial mediation pattern aligns with Bowen’s (1978) family systems theory, which conceptualizes self-differentiation as an important but not exhaustive component of psychological functioning. Supporting evidence comes from Wang and Fu’s (2019) parallel research, which similarly identified both direct and indirect effects of attachment insecurity on career decision-making difficulties.

These results carry several important implications. First, they demonstrate that emotional and cognitive factors interact in complex ways to shape career decisions. Second, they reveal that attachment characteristics influence vocational choices through multiple concurrent mechanisms. Third, they highlight how the relative importance of different pathways varies across cultural contexts. The observed attenuation of self-differentiation’s direct effects in our Chinese sample likely reflects the cultural prioritization of familial and social guidance over purely autonomous decision-making - a pattern well-documented in collectivist societies.

This cultural lens proves particularly valuable for understanding why traditional Western models emphasizing independent decision-making may require modification when applied to Chinese educational contexts. The findings suggest that career interventions in China should account for both individual psychological factors (like attachment security and self-differentiation) and the substantial influence of social networks and family systems on vocational development. Future research should explore these cultural variations more systematically, potentially through comparative studies across different societal contexts.

### 6.7 H6 supported: Social support partially mediates the effect of adult attachment on career decision-making

Social support partially mediates the relationship between adult attachment and career decision-making. Specifically, attachment anxiety and avoidance indirectly impair career decision-making by limiting access to social resources. Social support plays dual roles of “buffering” and “transforming” in this pathway, aligning with Cohen and Wills’ (1985) stress-buffering hypothesis. Emotional encouragement and instrumental resources provided by social support alleviate decision-making anxiety caused by attachment insecurity. Individuals with high social support may even transform attachment anxiety into decision-making motivation. However, the partial mediation suggests that social support alone cannot fully counteract the negative effects of insecure attachment; simultaneous cultivation of emotional regulation skills is necessary to collectively aid decision-making.

Although social support serves as a mediator in the chain pathway linking adult attachment to career decision-making, its independent mediating role remains significant. This highlights its critical compensatory value for individuals with attachment anxiety or avoidance, as external support can mitigate the impact of insecure attachment. These findings extend Lent et al. (2000) social cognitive model by emphasizing the compensatory role of external social resources in emotional regulation. In China’s collectivist context, where decisions are often relationship-oriented and career choices are shaped by collective input, social support reduces decision-making risks through shared responsibility. This underscores the unique mediating value of social support in Chinese cultural groups.

### 6.8 H8 partially supported

Among demographic variables, significant moderating effects were found for academic year, but not for gender, discipline, or education level. The chain mediation effect was applicable to students in grades 1–3 but disappeared among fourth-year students. This reflects stage-specific characteristics in career decision-making mechanisms. First- to third-year students, in the exploration phase, rely heavily on psychological adjustment (self-differentiation) and social support to develop career interests and values. In contrast, fourth-year students, transitioning to employment or internships, prioritize practical factors like salary and work environment, diminishing the influence of psychosocial mechanisms. This aligns with Super’s (1980) career development theory, which posits that career decisions involve balancing multiple life roles.

For students in grades 1–3, whose primary role is academic, social support helps reconcile conflicts between studies and career exploration, facilitating decision-making. Fourth-year students, however, shift focus to workplace roles, where competitive pressures prioritize immediate practical concerns over psychological factors. These findings provide a basis for stage-specific career interventions: lower-grade students may benefit from psychological empowerment, while upper-grade students require enhanced access to occupational resources.

The non-significant gender difference suggests convergence in gender roles among Chinese university students, with both males and females equally valuing social support, consistent with Li and Kato’s (2006) findings. The lack of discipline- or education-level effects may stem from standardized career education and guidance systems in Chinese universities, which lack tailored support for specific disciplines or academic tiers. This highlights the need for discipline-industry-aligned career services to address gaps in generalized approaches.

## 7 Study limitations

### 7.1 Geographical and population constraints

The data for this study were collected exclusively from undergraduate students at three universities in Shandong Province. Although institutional diversity (comprehensive, normal, and medical universities) was controlled, China’s significant regional disparities and the exclusion of vocational college students, graduate students, and working professionals limit generalizability. Future studies should incorporate samples from more provinces and diverse educational backgrounds (e.g., vocational students, graduate students, and professionals) to validate the model’s applicability across populations.

### 7.2 Reliability issues in measurement tools

The Emotional Cutoff (EC) subscale of the Differentiation of Self Inventory exhibited low reliability (*α* = 0.645), potentially due to insufficient cultural relevance of certain items. For example, the item “I avoid sharing my inner feelings with others” may be misinterpreted in China’s collectivist context as “maintaining group harmony” rather than “emotional avoidance.” Subsequent research could refine item wording (e.g., “I prefer to manage emotional issues independently”) or increase the number of items to enhance reliability and cultural appropriateness.

### 7.3 Cultural generalizability requires verification

The proposed chain mediation model is rooted in China’s collectivist cultural context. Its mechanisms may differ in individualistic cultures—for instance, Western students might rely more on self-efficacy than familial support during career decision-making. Cross-cultural comparative studies (e.g., samples from China, the U.S., and Germany) could clarify how cultural contexts moderate pathway coefficients and define the model’s universal applicability.

### 7.4 Future research directions

Although this study has revealed the complex mechanisms through which adult attachment, self-differentiation, and social support influence career decision-making, there are still some limitations. The study’s cross-sectional design limits examination of temporal dynamics in these relationships. Longitudinal approaches could better reveal how attachment, self-differentiation, and social support interact developmentally across career transitions. Future research should also examine additional factors like psychological resilience and academic stress to provide a more complete picture of career decision-making mechanisms.

## 8 Conclusion and implications

### 8.1 Conclusion

The study demonstrates that adult attachment exerts both direct and mediated effects on career decision-making processes. Analysis reveals that individuals with insecure attachment patterns experience significantly greater vocational indecision and anxiety when confronting career choices, characterized by chronic hesitation and impaired confidence in decision outcomes. These findings align with attachment theory’s proposition that early relational experiences shape subsequent approach/avoidance tendencies in important life decisions.

Regarding self-differentiation, the results indicate a consistent positive association with career decision-making competence. Highly differentiated individuals exhibit three distinct advantages in vocational decision contexts: (1) enhanced emotional regulation capacity when evaluating career options, (2) greater cognitive clarity in assessing personal vocational fit, and (3) increased autonomy in resisting external pressures during the decision-making process. This pattern supports Bowen’s conceptualization of self-differentiation as a critical psychological resource for navigating complex life choices.

Social support directly influences career decisions. A good social support system—including the support of family, friends, classmates, and mentors—can provide emotional comfort and informational help to individuals, alleviating anxiety and confusion in career decisions and helping them make informed choices.

Adult attachment also influences career decision-making through the mediating role of self-differentiation and social support on a continuous basis. That is, individuals with insecure attachment are more likely to be emotionally dependent on others and find it difficult to make independent decisions due to their lower level of self-differentiation. Nonetheless, with the help of social support systems, they can obtain external support for decision-making, thereby alleviating the negative impact of attachment insecurity on career decision-making.

The chain mediation model had good explanatory power and prediction ability on randomly selected samples, and the model had universality and applicability, which can better explain the career decision-making process. Except for grades, there were no significant differences in the interrelationships between adult attachment, self-differentiation, social support, and career decision-making among college students of different genders, educational levels, and disciplines. These results suggest that these psychological mechanisms are prevalent in a wide range of college students.

### 8.2 Implications

#### 8.2.1 Emphasis is placed on the influence of emotional factors on career decisions

In the career decision-making process, college students are not only influenced by career information and realistic conditions; emotional and attachment styles also play a key role. Students—especially those with insecure attachments—may show more anxiety and hesitation in career choices due to a lack of emotional independence. Therefore, in career counseling, special attention should be paid to students’ emotional states and attachment patterns to help them better understand and deal with emotional problems so as to improve the rationality and efficiency of their career decision-making.

#### 8.2.2 Enhance career decision-making ability by improving the level of self-differentiation

Improving the level of self-differentiation in college students is another effective way to help them improve their career decision-making ability. Students with higher self-differentiation abilities are able to maintain clearer thinking and emotional independence when facing career choices and are not easily affected by external interference or emotional fluctuations. Therefore, vocational and mental health education should focus on improving students’ emotional regulation and self-awareness skills, helping them to achieve more independent and autonomous decision-making.

#### 8.2.3 Build a multi-dimensional social support system

Social support plays an essential role in relieving the stress of career decision-making and improving career choices. Schools, families, and society should work together to provide students with a broad network of support, including emotional comfort, informational guidance, and resources for career development. For example, schools can provide students with more career guidance and emotional support through mentorship, career counseling services, and alum networks to help them successfully navigate the critical stages of career decision-making.

#### 8.2.4 A career coaching model integrating attachment, self-differentiation, and social support

Our study reveals the complex relationships between adult attachment, self-differentiation, and social support. Therefore, in actual career counseling, these three should be integrated, and multi-level interventions should be designed. For students with insecure attachment, counselors can mitigate the adverse effects of emotional dependence on career decision-making by helping them improve their self-differentiation skills and guiding them to build and utilize social support networks. These initiatives can help students better cope with the emotional pressures of career choices and make more rational and mature career decisions.

#### 8.2.5 Construct intervention strategies with a wide range of applicability

Our study found that the mechanisms of adult attachment, self-differentiation, and social support on career decision-making were universal, regardless of students’ gender, educational level, and subject area. Therefore, career counseling and educational intervention strategies can be applied to a broad population of college students, and there is no need to make significant distinctions based on the background characteristics of individuals. Career counselors and educators can provide similar services, such as emotional support, self-differentiation, and social support-building for all students.

However, establishing a phased career intervention framework is essential. For first-to third-year undergraduates, interventions should focus on psychological empowerment through group counseling, self-differentiation training courses (e.g., emotion management, boundary-setting), and social support network building (e.g., mentorship programs, peer support groups). These initiatives aim to cultivate emotional regulation skills and resource integration awareness.

For fourth-year students and beyond, interventions should shift toward practical resource provision, including industry-aligned internships, corporate site visits, job skill workshops, and the integration of career decision-making tools (e.g., decision balance sheets, occupational databases) to help students translate psychological resources into actionable outcomes.

Additionally, career guidance and intervention services for university students should prioritize discipline-industry collaborative initiatives. Although discipline differences did not significantly moderate the pathways, standardized career support may overlook discipline-specific needs. Universities are advised to partner with industries to develop discipline-specific career resources (e.g., hospital-university joint training programs for medical students, industry-academia collaboration projects for engineering students), thereby strengthening direct alignment between professional competencies and labor market demands.

In conclusion, the study shows that career decision-making is not only a process of information collection and analysis but also has a profound impact on emotional factors, self-differentiation levels, and social support systems. Therefore, career counseling and education should comprehensively consider these psychological factors and provide comprehensive support and counseling to help college students make more rational and mature career choices.

## Data Availability

The original contributions presented in the study are included in the article/supplementary material, further inquiries can be directed to the corresponding author.
